# The effect of acupressure on pain level and hemodynamic parameters after coronary angiography: a randomized controlled study

**DOI:** 10.3389/fcvm.2023.1173363

**Published:** 2023-07-14

**Authors:** Barış Düzel, Tuğba Çam Yanik, Canan Kanat, Gülay Altun Uğraş

**Affiliations:** ^1^ Department of Cardiology, Mersin City Training and Research Hospital, Mersin, Türkiye; ^2^ Faculty of Nursing, Department of Surgical Nursing, Mersin University, Mersin, Türkiye

**Keywords:** acupressure, angiography, hemodynamic parameters, pain, coronary

## Abstract

**Background:**

Subsequent to coronary angiography, procedures performed to prevent bleeding may cause pain in the patient. In this study, we aimed to determine the effect of acupressure on pain level and hemodynamic parameters in patients undergoing coronary angiography.

**Method:**

In this prospective, a two-arm (1:1), randomized controlled trial was conducted, with 124 patients undergoing coronary angiography included. The randomly assigned study group (*n* = 62) received acupressure on the LI4 (on the dorsum of the hand, between the 1st and 2nd metacarpal bones), PC6 (three fingers above the wrist), and LI11 (at the lateral end of the transverse cubital crease) points for 15 min 2 h after angiography, while the control group (*n* = 62) received no acupressure. Data were collected using the visual pain scale (VAS) and hemodynamic parameters [systolic blood pressure (SBP), diastolic blood pressure (DBP), heart rate (HR), respiratory rate (RR), and peripheral oxygen saturation (SpO_2_)], monitoring form before, immediately after, and at 10, 20, and 30 min after acupressure.

**Results:**

In the study, it was found that patients had moderate pain after coronary angiography (study group: 5.02 ± 2.27; control group: 3.98 ± 1.82). When the groups were compared, it was found that the VAS score of the study group before angiography was significantly higher than that of the control group, but lower than the control group immediately after acupressure, and at 10, 20, and 30 min after acupressure. In addition, it was determined that acupressure was significantly higher in DBP and RR in the study group compared to the control group at 20 and 30 min; it was not effective in terms of SBP, HR, and SpO_2_ values.

**Conclusion:**

The results of the study indicated that patients reported moderate pain after coronary angiography, and that acupressure was effective in reducing the pain level, but affected only the DBP and RR hemodynamic parameters. Since the study was single-centered and followed for a short time, it is recommended to conduct new studies with a longer duration.

## Introduction

Coronary artery disease is the most common cause of cardiovascular disease (1), which ranks first among the causes of death worldwide (2). Coronary angiography (CA), the gold standard for the diagnosis and treatment of coronary artery disease (CAD), may cause pain because it is an invasive procedure and various applications are performed to control bleeding after the process. Following CA, sandbags or pressurized dressings applied to the intervention area to prevent bleeding may cause pain in the patient (3). As a result of the neuroendocrine effect of pain, an increase in heart rate (HR), respiratory rate (RR), and blood pressure and oxygen demand of the heart may be observed. This may increase the workload of the heart, the risk of complications, and may negatively affect the hospitalization process. It is important to implement pain-reducing interventions to prevent pain-related complications experienced by patients after CA (3, 4). In addition to pharmacologic methods, such as non-opioid analgesics, non-steroidal anti-inflammatory drugs, and opioid analgesics, nonpharmacologic methods can also be used to reduce pain (5, 6). The fact that pharmacologic methods have undesirable side effects such as hypotension, tachycardia, respiratory depression, constipation, nausea, and vomiting has led to an increase in the use of nonpharmacologic methods (aromatherapy, massage, acupuncture, acupressure, etc.) for treatment of pain control (4, 7, 8). Acupressure, one of these methods, is a body-based practice based on traditional Chinese medicine. Acupressure is an application that ensures the proper functioning of energy channels by applying pressure to acupuncture points on the meridians carrying energy in the body with fingers, hands, elbows, or special stimulating bands. The pressure applied to acupressure points initiates the process of nociception. The impulses stimulate serotoninergic and enkephalinergic neurons and the analgesic system is activated. Acupressure increases the secretion of endorphins, a neurochemical that has a pain-relieving effect, reduces tension in the muscles, increases the transmission of endorphins and serotonin by reducing the amount of cortisol, and increases the amount of oxygen in the blood circulation, thus providing relaxation in individuals. In addition, according to the gate control theory, the pressure applied during acupressure stimulates large diameter fibers, closing the door to small diameter fibers that carry pain. Therefore, the transmission of pain impulses to the brain is prevented by closing the nerve vessels. In the control of pain, studies have aimed to reduce the amount of analgesic use and stabilization of hemodynamic parameters by reducing the pain level through acupressure application (3, 5, 6, 9, 10). Since acupressure is a noninvasive, side-effect-free, safe, easy-to-apply, and effective method, its use is becoming increasingly widespread (3–5). In the literature, it has been reported that the most commonly used acupressure points in pain management are LI4, LI11, and PC6 (5, 6, 11, 12). Narimani et al. (2018) reported that acupressure applied to the LI4 point decreased the pain level immediately following the acupressure, and sustained its effect for 20 min after coronary artery by-pass graft surgery (5). In another study, it was reported that the pain levels of patients who applied acupressure after CA were lower 4, 6, and 8 h after the implementation, compared to those who did not receive acupressure (*p* < 0.05) (6). Numerous studies in the literature detail the effect of acupressure on pain level following CA (3, 6, 11, 12–14). In a limited number of studies, it was stated that acupressure was applied within 24 h or more than once, and the immediate effect of acupressure applied once was examined (11, 12, 15–18). In previous studies, the LI4, LI11, and PC6 points were found to be the most commonly used points to reduce the pain level (11, 12, 15–18); however, no study has used them together or examined their effects on pain and hemodynamic parameters after CA. Therefore, the aim of this study was to determine the effect of acupressure, which is one of the nonpharmacologic methods, on pain and hemodynamic parameters [systolic blood pressure (SBP), diastolic blood pressure (DBP), peripheral oxygen saturation (SpO_2_), RR, and HR] in patients after CA. One of the hypotheses of this study is that acupressure used in patients affects pain levels. The other hypothesis of this study is that acupressure used in patients affects hemodynamic parameters.

## Materials and methods

### Study design and sample

This study was conducted as a prospective parallel two-arm [1:1] randomized controlled experimental study. The study sample consisted of 124 patients who underwent CA in a public hospital between 25 November 2022 and 30 December 2022. Taking the study of Vagharseyyedin et al. (2022) as a reference (3), as a result of the power analysis performed in G*Power 3.1.9.2 trial version with an effect size of at least 0.535 units for the difference between the mean pain scores between the two groups, a total number of 112 patients, 56 in each group, was determined with 80% power and a maximum two-way 5% type 1 error. The dropout rate was taken as 10% and added to the number calculated; the total sample number was found to be 124 so that the number of patients in both groups was equal. The study was completed with 124 patients who underwent CA (experimental group = 62; control group = 62). A Clinical Trials registration (NCT05486533) was completed and the study was carried out without any changes in the protocol.

### Inclusion and exclusion criteria

**Figure 1:**
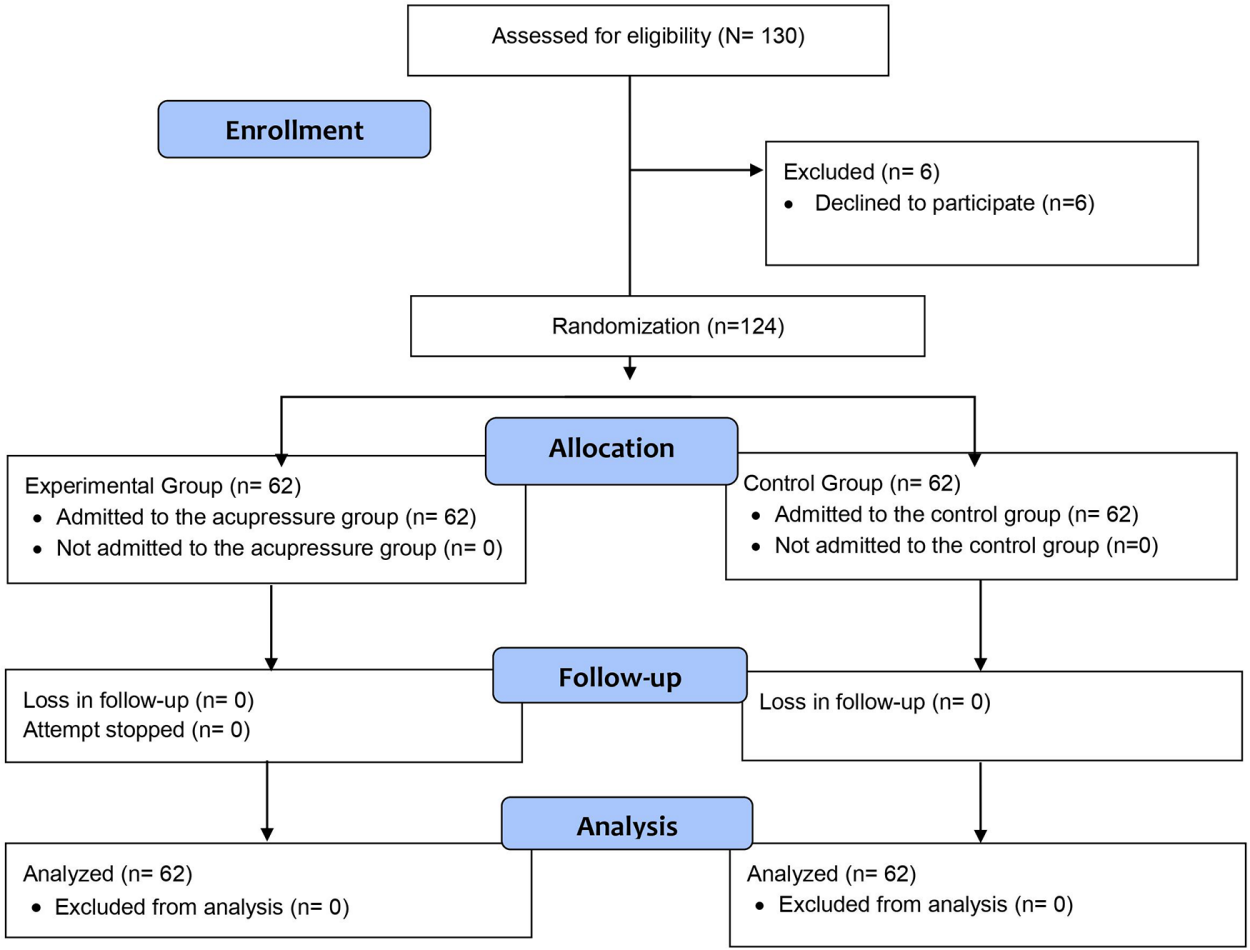
CONSORT flowchart of the research.

The study included patients who underwent CA, who voluntarily agreed to participate in the study, who had no physical problems that would prevent the application of acupressure to the LI4, PC6, and LI11 points, who were over 18 years of age, whose general condition was stable, who had stable CAD without myocardial infarction or acute ischemic heart disease, who had no previous acupressure experience, who did not receive sedative, tranquilizer, or opioid agents 5 h before and after CA, who were not planned for emergency CA, who underwent femoral artery intervention, who did not have chronic pain, who were not diagnosed with COVID-19 during the study, and who did not have any psychiatric diagnosis. During the study, 130 patients underwent CA. Six patients who did not meet the inclusion criteria were excluded because they did not want to participate (Figure 1).

### Randomization and blinding

In the study, a total of 124 patients were randomly assigned to groups A and B by the same statistician using the block randomization method. As a result of the draw of lots, it was determined at the beginning of the study whether group A and B would be the experimental or control group. The information showing that the patients included in the study sample were assigned to groups A and B according to the randomization table was placed in an opaque envelope and this envelope was kept by the coordinating investigator (GAU). Compliance of the patients with the inclusion criteria was determined by the cardiologist researcher (BD). When the researcher (TÇY) attended the patient for application of acupressure, he opened the envelope and learned which group the patient was in. Since acupressure was to be applied only to the patients in the experimental group, the patients who received and the researcher (TÇY) who applied acupressure could not be blinded due to the nature of the study. The researchers (BD and CK) who collected the data before and after the application were blinded because they did not know which group the patients were in. In addition, when the study was completed, the data were transferred to a computer by a statistician who did not know about the A and B groups; the data were analyzed, and the findings were reported by a statistician independent of the study. Thus, the data analysis and statistics phase was also blinded.

### Outcome measures and instruments

The primary outcome measure of the study was the effect of acupressure application on patients' pain level. The secondary outcome measure of the study was the effect of acupressure application on patients' hemodynamic parameters (changes in SBP, DBP, HR, RR, and SpO_2_).

Data were collected using the “Descriptive Characteristics Form,” “Visual Analog Scale,” and “Hemodynamic Parameters Monitoring Form”.

***Descriptive Characteristics Form***: The Descriptive Characteristics Form, which was developed by the investigators in line with the literature, included a total of six questions evaluating the sociodemographic characteristics (age, gender, marital status, educational status, etc.) of the patients (12, 15–18).

***Visual Analog Scale (VAS):*** The VAS consists of a 10 cm long line with subjective descriptive statements at both ends of the scale (0 cm = absent and 10 cm = extremely present) (19). The individual places a mark at the appropriate place on this 10 cm line on the scale. The pain level of the individual is determined numerically in cm by measuring with a ruler the distance from the beginning of the scale to the place where the individual places the mark. A score of 0 obtained from the VAS indicates no pain, 1–3 points indicate mild pain, 3–6 points indicate moderate pain, 7–9 points indicate severe pain, and 9–10 points indicate the maximum possible pain level (15, 19).

***Hemodynamic Parameters Monitoring Form***: The Hemodynamic Parameters Monitoring Form was prepared to record the values of SBP, DBP, HR, RR, and SpO_2_ of the control and experimental group patients before, immediately after, and at 10, 20, and 30 min after the intervention.

### Interventions

Two hours after CA, patients in the experimental and control groups were first asked to sign an informed consent form about the study. Afterwards, the Descriptive Characteristics Form, VAS, and Hemodynamic Parameters Monitoring Form were completed.

In the clinic, 10 cc prilocaine hydrochloride local anesthetic drug was administered subcutaneously to the femoral region before CA. Considering that the half-life of this drug is on average 2–4 h (20), and as a result of the observations, since patients usually started to complain of pain 2 h after CA, acupressure was applied at the 2nd hour after CA before the pain intensified. When the patient complained of pain in the clinic after CA, analgesics (Paracetamol, 10 mg, IV) were administered if necessary. In the literature, it was found that the application of acupressure to a single point between 0 and 2 h postoperatively was not effective in reducing the pain level (21). This is why here, three different points that were effective for pain control (22, 23) were selected for acupressure in order to make the application more efficient in terms of pain management. All of the patients in our study were not routinely given any analgesic after the CA procedure.

**Figure 2:**
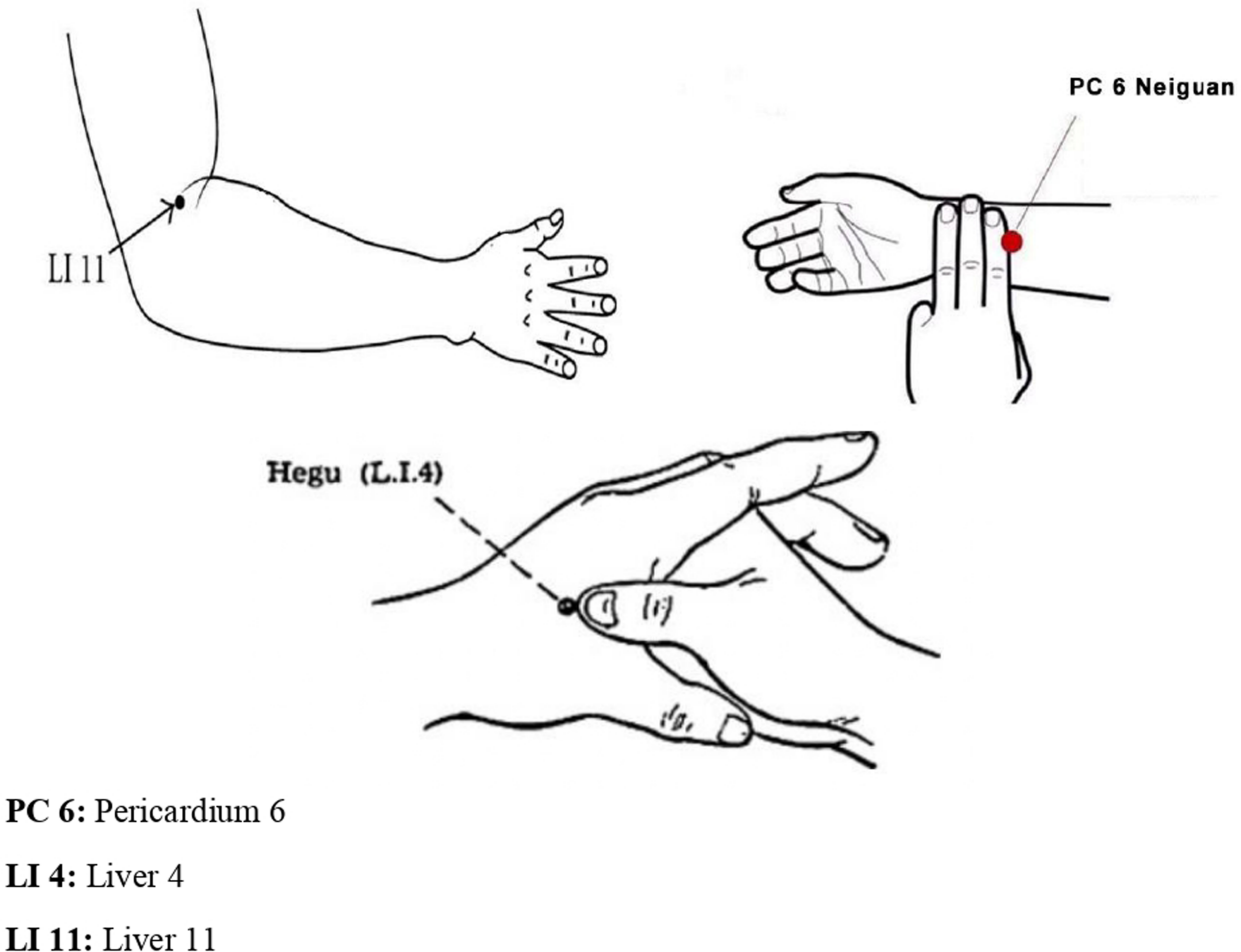
Acupressure points in the study (19–21).

Acupressure points [LI4 (on the dorsum of the hand, between the 1st and 2nd metacarpal bones), PC6 (three fingers above the wrist), and LI11 (at the lateral end of the transverse cubital crease)] (Figure 2) (22–24) were marked with an acetate pen by a certified researcher (T.Ç.Y.) in the experimental group (12, 16, 18). The “cun” measurement unit was used to locate the points where the application would be performed. The unit distance of one cun was converted to centimeters (cm) for each individual by measuring their thumb width in cm. The order of use of the points was determined as LI4, PC6, and LI11, first right and then left. Before starting the application, the area to be pressurized was gently rubbed with the palm of the hand for 20–30 s to reduce sensitivity. The identified point was pressurized with the thumb, index, or middle finger for 5 s to a depth of 1 cm–1.5 cm. Consecutive pressure applied to each acupressure point was continued for 2 min after resting for 2 s. Successive pressures were applied at a frequency that does not disturb the individual, does not cause pain, and has a calming effect (25–27). The patients in the study group were treated at the LI4, PC6, and LI11 points (six points in total) for an average of 15 min, for 2 min at each point. Since acupressure was performed by the same researcher (TÇY) in all patients, the application was standardized. Communication was maintained with the patients during the application against possible negative conditions; no negative feedback was received. There were no adverse events in the patients during and after acupressure.

The control group was not provided with any treatment other than routine treatment and care.

In the experimental group, VAS assessments were performed 2 h after CA, immediately after acupressure application, and at 10, 20, and 30 min after the application. Assessments were performed in the control group at the same times as in the study group (2 h after CA, 10, 20, and 30 min after the application in parallel with the acupressure group times). At the specified times, SBP, DBP, HR, RR, and SpO_2_ values were evaluated with a portable monitor (Drägerwerk, Model A MS32996, Germany) and recorded on the Hemodynamic Parameters Monitoring Form.

### Statistical analyses

Descriptive statistics were expressed as frequency, percentage, mean, standard deviation, and maximum-minimum. Categorical variables were analyzed by Fisher–Exact, Continuity Correction, and Pearson-*χ*2 tests. Compliance of the variables with normal distribution was performed using the Shapiro–Wilk test. An independent samples *t*-test was used for intergroup comparisons of data conforming to normal distribution; a Mann–Whitney *U* test was used for data not conforming. The significance level of statistical tests was *p* < 0.05 in all analyses.

## Results

The patients in the experimental and control groups were similar in terms of descriptive characteristics (*p* > 0.05) (Table 1).

**Table 1:** Comparison of descriptive characteristics of patients (*n* = 124).

Characteristics	Control group x¯ ± SS		Experimental group x¯ ± SS		Test value	*p*
Age* (Year) (Min: 37 Max: 85)	59.35 ± 10.29		59.73 ± 10.75		0.196	0.845
Gender**	n	%	n	%		
Female	26	41.9	29	46.8	2.065	0.151
Male	36	58.1	33	53.2		
Marital status**						
Single	11	17.7	8	12.9	0.249	0.618
Married	51	82.3	54	87.1		
Education status**						
No reading or writing	9	14.5	12	19.4	2.640	0.451
Primary education	31	50	34	54.8		
Secondary education	18	29	15	24.2		
High education	4	6.5	1	1.6		
Chronic disease status**						
No	24	38.7	17	27.4	1.312	0.252
Yes (One or more)	38	61.3	45	72.6		
*Hypertension*	28		30			
*Diabetes*	*18*		*18*			
*COPD*	*4*		*4*			
*Coronary artery disease*	*11*		*11*			
Number of applied angiography**						
*1*	41	66.1	33	53.2	3.557	0.469
*2*	9	14.5	17	27.4		
*3*	6	9.7	7	11.3		
*4*	5	8.1	4	6.5		
*5*	1	1.6	1	1.6		

* Independent groups *t* test was used in data analysis.

** Chi-square test was used in data analysis. COPD, chronic obstructive pulmonary disease.

### Primary outcome: comparison of pain level

In the study, it was found that the pain levels of the patients in the experimental (5.02 ± 2.27) and control groups (3.98 ± 1.82) were moderate at the 2nd hour after CA (before acupressure). The pain levels of patients in the experimental and control groups were compared; the average patients pain score the in the study group before the application was significantly higher than that in the control group, and immediately after the application, the average of the VAS scores 10, 20, and 30 min after the application was significantly lower (*p* < 0.05) (Table 2).

**Table 2:** Comparison of Patients’ visual analog scale scores and hemodynamic parametres (*n* = 124).

	Measurement time	Control group x¯ ± SS	Experimental group x¯ ± SS	Test	*p*
Visual analog scale	Before intervention	3.98 ± 1.82	5.02 ± 2.27	2.795*	**0**.**006**
	Immediately after intervention	4.03 ± 1.83	3.18 ± 1.61	−2.759**	**0**.**007**
	10 min after intervention	3.53 ± 1.70	2.45 ± 1.56	−3.688*	**0**.**000**
	20 min after intervention	3.05 ± 1.84	1.61 ± 1.23	−5.101*	**0**.**000**
	30 min after intervention	2.87 ± 1.88	1.10 ± 1.04	−6.516*	**0**.**000**
Systolic blood pressure	Before intervention	137.55 ± 19.20	133.08 ± 26.28	−0.990**	0.322
	Immediately after intervention	133.52 ± 19.50	130.97 ± 20.74	−0.705*	0.482
	10 min after intervention	130.47 ± 18.00	130.79 ± 19.14	0.097*	0.923
	20 min after intervention	129.61 ± 16.05	133.68 ± 15.63	1.428*	0.156
	30 min after intervention	127.89 ± 17.70	130.42 ± 16.87	0.815*	0.416
Diastolic blood pressure	Before intervention	76.71 ± 9.81	75.45 ± 11.70	−0.649*	0.518
	Immediately after intervention	75.37 ± 8.93	75.66 ± 11.26	−0,065**	0.948
	10 min after intervention	75.53 ± 9.92	74.44 ± 11.15	−0.579*	0.564
	20 min after intervention	74.46 ± 7.51	78.27 ± 8.44	−2.585**	**0**.**010**
	30 min after intervention	73.54 ± 8.59	78.26 ± 8.55	3.060*	**0**.**003**
Heart rate	Before intervention	71.10 ± 10.93	71.97 ± 14.77	−0.510**	0.610
	Immediately after intervention	70.58 ± 11.64	70.37 ± 14.65	−0.963**	0.336
	10 min after intervention	70.51 ± 11.02	70.97 ± 13.56	−0.553**	0.580
	20 min after intervention	70.66 ± 11.02	73.68 ± 12.77	1.276**	0.202
	30 min after intervention	70.48 ± 10.75	73.98 ± 12.30	1.7,727**	0.076
Respiration rate	Before intervention	20.16 ± 2.09	20.58 ± 1.86	1.180*	0.240
	Immediately after intervention	19.90 ± 1.90	19.81 ± 1.61	−0.065*	0.948
	10 min after intervention	19.90 ± 1.71	19.88 ± 1.49	−0.056*	0.956
	20 min after intervention	20.03 ± 1.90	21.10 ± 1.80	3.205*	**0**.**002**
	30 min after intervention	19.90 ± 1.72	21.23 ± 1.79	4.204*	**0**.**000**
Peripheral oxygen saturation	Before intervention	96.23 ± 1.03	95.92 ± 1.42	−1.377*	0.171
	Immediately after intervention	95.97 ± 1.07	96.27 ± 1.37	1.388*	0.168
	10 min after intervention	96.06 ± 1.11	96.03 ± 1.31	−0.148*	0.883
	20 min after intervention	96.18 ± 1.11	96.24 ± 1.20	0.311*	0.756
	30 min after intervention	96.06 ± 1.01	96.21 ± 1.15	0.749*	0.455

* Independent groups *t* test was used.

** Mann-Whitney *U* test was used in independent groups.

Bold values significant *p* < 0.05.

### Secondary outcome: comparison of patients' hemodynamic parameters

When the hemodynamic parameters of the patients in the experimental and control groups were compared, there was no difference observed between the groups for the parameters SBP, HR, and SpO_2_, but there was a difference for DBP and RR (*p* > 0.05). The DBP and RR values of the patients in the experimental group were found to be significantly higher than those of the control group 20 and 30 min after the application (*p* < 0.05) (Table 2).

## Discussion

In this research, in which the effect of acupressure on the pain level and hemodynamic parameters of patients after CA was investigated, it was observed that patients experienced moderate pain after CA and that acupressure maintained its effect in reducing the pain level for 30 min, but did not affect other hemodynamic parameters except for DBP and RR.

### The effect of acupressure application on pain

Coronary angiography itself, with painful applications such as sandbags or pressurized dressing, applied to the venture area to prevent bleeding, can increase the level of pain by affecting the perception of pain (13, 14). In previous research, it was reported that an increase in pain level was expected in the hours after CA (3), and that the pain levels of patients were high/moderate (6). In this study, similar to the previous study, it was observed that all patients had moderate pain after CA. Although it was found that the patients in the experimental group experienced more pain than patients in the control group before the application, it was determined that this pain decreased significantly after acupressure until the 30th minute after the application. The decrease in pain in patients in the experimental group can be explained by the endorphin response; the acupressure applied provides relaxation in the individual as a result of muscle relaxation and increased blood flow. In addition, the closure of nerve gates (gate control theory) with the application to the pressure points may have reduced pain by preventing pain impulses from reaching the brain (6). Similar to this study, acupressure has been reported to be effective for pain control in previous studies (6, 11). In contrast to the results of the present study, in the study of Kılınç and Karaman Özlü, the authors found that acupressure was not effective for reducing the pain level after laparoscopic surgery; however, acupressure was performed using “acupressure application tape” (21). In our study, the application of acupressure to the patient using the touch of a health professional, with similar results to previous studies, suggested that it was effective in reducing the level of pain (11, 28).

In this research, acupressure was applied to the hand (LI4), arm (PC6), and elbow bend (LI11) points. In previous studies, acupressure was applied to different points on the hand [LI4 (5, 12, 13, 15–17), LI2 (12), and SI3 (6)]; wrist (HT7) (11, 15–18); different points on the arm [PC6 (12, 18) and LI11 (16, 17)]; ear (auricular) (3, 13); face (GV26) (6); different points on the leg (GB34, GB37 (12), and ST36 (6); foot (GB42) (12); ankle (BL60); and chest (GB24) (12), which were reported to be effective in controlling pain after different interventions. In this study, the endorphin theory, activated by pressure applied to three different acupressure points, may have been effective in reducing the pain level of the patients. The pressures applied in line with this theory may have activated the central nervous system and initiated the nociception process, resulting in an increase in endorphin, serotonin, norepinephrine, and enkephalin levels in the plasma, and may have produced an analgesic effect (29, 30). In addition, when these pressure points are explained using the gate control theory, which is also supported by the literature (5, 11, 15–17), with continuous stimulus created by the pressure applied to each point for 2 min, large diameter fibers may have caused a decrease in the pain level of the patients by carrying the stimulus and closing the doors to painful stimuli (10, 30). These results show that acupressure is effective in the control of pain, regardless of the point at which acupressure is applied, and that it can also help to reduce the risk of coronary microvascular dysfunction in patients (31–33). These results confirm our hypothesis that acupressure used in patients affects pain levels.

### The effect of the acupressure application on hemodynamic parameters

Pain that occurs after invasive interventions such as CA (3) causes stimulation of the hypothalamus and an increase in sympathetic nervous system activation. As a result of stimulation of the pituitary gland by the hypothalamus, reabsorption of water from the kidneys occurs and blood pressure increases. Epinephrine and norepinephrine, secreted as a result of activation of the sympathetic nervous system, increase the HR and oxygen demand of the heart. When pain is not controlled or reduced, changes in hemodynamic parameters may lead to the development of complications (30, 34). Acupressure application reduces/prevents the access of pain to the brain and relaxes the body via an endorphin response. Reducing the effect of pain on the hemodynamic parameters and keeping these parameters at normal value intervals can prevent complications that may occur (9).

In the study, it was determined that acupressure produced significantly higher DBP and RR values at the 20th and 30th min in the experimental group compared to patients in the control group; however, it had no significant effect on SBP, HR, or SpO_2_ values. In addition, the hemodynamic parameters of all patients in the study were within normal ranges. In the literature, a study examining the effect of acupressure on hemodynamic parameters before CA was found (25), but no study examining its effect after CA was found. Mei et al. (2017) applied acupressure for a total of 15 min at three different acupressure points (EXHN5, GB20, and HT7) once a day for three days to patients undergoing CA and found that acupressure had no effect on hemodynamic parameters. In this study, similar results were obtained (25), except for the increase in DBP and RR values. In another study, acupressure was applied to the LR3 (Taichong) point for 3 min in patients with hypertension. In the study, compared to the sham acupressure group, it was stated that SBP and DBP decreased after application in the group where acupressure was applied to the real points (*p* < 0.05). In contrast to our study, in the aforementioned study, it was found that blood pressure values decreased immediately after acupressure application, at 15 and 30 min (35). In contrast to this study, in a study in which five different acupressure points (LU1, LU9, LI4, PC6, ST36) were treated in mechanically ventilated patients for an average of 20 min, it was determined that the RR of all patients was low at the beginning. Immediately after the application and at 30 min, it was found that the RR of the acupressure group increased to the desired level compared to the other group (*p* < 0.05) (36). In another study, acupressure was applied to patients with acute myocardial infarction at five different points (PC6, HT7, SP6, EX3, EX5) for an average of 18 min for two days before going to sleep at night. In contrast to this study, the authors found that HR decreased in the acupressure group compared to the control group (*p* < 0.05) (37). In the study by Batvani et al., acupressure was applied to five different acupressure points, twice a day for 2 min each for three days in patients with myocardial infarction. The researchers found that the O_2_ value of the acupressure group increased compared to the control group, contrary to our study (*p* < 0.05) (38).

The emergence of a feeling of relaxation due to the completion of a stressful invasive procedure such as CA in all patients and the decrease in pain over time after the procedure (10) may have caused the body to limit the neuroendocrine response. This may have led to minor changes in the hemodynamic parameters; they may have remained within the normal range. In addition, the analgesic effect of acupressure, caused by the activation of the gate control and endorphin theories, provides relaxation to the patients. This relaxation may have helped to stabilize the hemodynamic parameters of the patients during the research period (29, 30). The results of this study reveal that acupressure applied after CA effectively reduces pain and has no effect on the improvement of hemodynamic parameters in this process. The lack of effect of acupressure on hemodynamic parameters in this study may be due to the fact that fewer points were used and application was performed only once compared to previous studies (36–38). In addition, the fact that the patients in this study were pressurized for a shorter time (2 min) than Lin et al. (2016) used may have caused no change in the blood pressure values (35). On the other hand, the lack of studies examining the effect of acupressure on hemodynamic parameters after CA prevents a comprehensive discussion of these results. These results confirm our hypothesis that acupressure used in patients only affects the values of the hemodynamic parameters DBP and RR.

## Conclusion

The results of this research showed that patients experienced moderate pain after CA and that acupressure applied by healthcare professionals was effective in controlling pain, DBP, and RR values. It may be recommended that acupressure, which is easy to apply, inexpensive, reliable, and has no side effects, should be applied in patients after CA. The fact that only one study was found in the literature in which acupressure was applied after CA shows that more comprehensive, multicenter, prospective, randomized, and controlled studies on this subject are needed.

### Strengths and limitations of the study

The most powerful aspect of this research is that it is the first study to determine the effect of acupressure on pain and hemodynamic parameters after CA. On the other hand, the discussion of the changes in hemodynamic parameters was therefore limited. In addition, since the study was single-centered, it cannot be generalized to all patients undergoing CA.

## Data Availability

The original contributions presented in the study are included in the article, further inquiries can be directed to the corresponding author.
